# Research progress and applications of gene activation editing technology in crops

**DOI:** 10.3389/fpls.2026.1787461

**Published:** 2026-03-20

**Authors:** Jing Lv, Xinxi He, Qi Wu, Yuhe Sun, Wenxuan Pu, Changbo Dai

**Affiliations:** 1 Tobacco Research Institute, Chinese Academy of Agricultural Sciences, Qingdao, China; 2 Technology Center, China Tobacco Hunan Industrial Co., Ltd., Changsha, China

**Keywords:** CRISPR/Cas, crop breeding, crop improvement, functional genomics, gene activation

## Abstract

In recent years, CRISPR/Cas gene editing technology has become a fundamental method in biological breeding. As a vital tool for overcoming technological obstacles, it is currently widely used in functional gene research and genetic enhancement across a variety of organisms. Currently, CRISPR activation (CRISPRa) technology based on dCas9 fusion transcription activation domains has emerged as a powerful tool for expanding the application of CRISPR/Cas systems in improving traits in plants, animals, and microorganisms. This overview starts by going over the underlying principles and components of gene activation editing technology, as well as the phases of development of its three generations. It summarises the present difficulties and potential directions in this field while concentrating on the use of gene activation editing in important crop traits including growth and development regulation, stress resistance, and quality regulation. The objective is to offer valuable insights for the research and development of crop breeding.

## 1 Introduction

Genome editing is a revolutionary technology that can precisely and specifically alter an organism’s genome (Pacesa et al., 2024). With the rapid advancement of genetic engineering, gene editing technology has evolved from zinc finger nucleases (ZFNs) and transcription activator-like effector nucleases (TALENs) to the CRISPR/Cas system based on clustered regularly interspaced short palindromic repeats (Yin et al., 2017). Among them, the CRISPR/Cas system has quickly become a vital instrument for enhancing the characteristics of microorganisms, animals, and plants due to its straightforward design, efficient operation, and powerful editing capabilities (Barrangou and Doudna, 2016; Zhu et al., 2020; Li et al., 2025). Utilizing the CRISPR/Cas system, a range of potent gene editing and regulation tools has been created through the optimization and modification of functional components. These include base editors (BE) (Gaudelli et al., 2017), prime-editor (PE) (Anzalone et al., 2020), CRISPR-based gene expression inhibition systems (CRISPRi), and CRISPR-based gene expression activation systems (CRISPRa) (Gilbert et al., 2014), significantly expanding the application scope of genome editing.

Despite the considerable promise of regulatory technologies like CRISPRa for enhancing crop traits, they encounter substantial obstacles regarding activation efficiency, targeting precision, and the management of off-target risks (Alariqi et al., 2025). With the deep integration of sequencing technologies, DNA synthesis techniques, and AI-assisted design, the precision and reliability of gene regulatory editing are expected to undergo systematic enhancement.This research provides a systematic review of the development of CRISPR/Cas-based gene activation regulation technology and its applications in improving important agronomic traits in crops. It also outlines future directions for this technology, aiming to offer theoretical references and technical insights for crop molecular design breeding.

## 2 Principles and components of gene activation editing technology

CRISPR activation (CRISPRa) is a transcriptional regulation technology based on the deactivated Cas protein (dCas). This method creates a dCas-activator complex by fusing a transcription activation domain to the dCas protein. This complex precisely targets the target gene’s promoter or enhancer regions under the guidance of sgRNA. By recruiting and stabilizing the transcription initiation complex, it achieves upregulation of target gene expression. Neither the genetic sequence nor DNA double-strand breaks are introduced during the entire procedure. CRISPRa, which mainly consists of the following elements, has been widely used in transcriptional regulation:

### 2.1 dCas protein

Targeted mutations in important nuclease domains cause dCas proteins, which are variations of Cas proteins, to lose their ability to cleave DNA. By altering the conserved RuvC and HNH nuclease domains, Cas proteins are able to maintain their capacity to bind DNA while losing their cleavage function when guided by gRNA (Moradpour and Abdulah, 2020). The RuvC domain is responsible for cleaving the non-target strand, with D10A being a common inactivating mutation;the HNH domain is responsible for cleaving the target strand, with H840A being a typical inactivating mutation. Qi et al. introduced a double mutant (D10A/H840A) into Streptococcus pyogenes Cas9 nuclease and found that DNA cleavage activity was completely eliminated, resulting in the formation of dCas9 (Qi et al., 2013). The dCas9 protein is used in the majority of current research, although it has been shown that the inactivated Cas12f1 protein can fuse with the transcription activation domain TV to jointly activate rice OsIPA1 expression (Wang et al., 2025). In terms of specific binding to target DNA sequences, the PAM recognition mechanism of dCas is identical to that of wild-type Cas proteins.

### 2.2 gRNA

Natural gRNA comprises two components: target-specific crRNA and auxiliary trans-activating crRNA (tracrRNA). Charpentier and Doudna fused the complementary regions of crRNA and tracrRNA to create a single guide RNA (sgRNA). sgRNA enhances Cas9 activity. Presently available engineered sgRNA sequences are roughly 96 nucleotides long and consist of one artificial tetraloop, a 20-nt target-complementary sequence (spacer), and a scaffold sequence that contains five secondary structural modules: the lower stem, upper stem, bulge, nexus, and hairpin (Briner et al., 2014). gRNA has three roles in CRISPRa: specific targeting: recognizes and binds to the target promoter/enhancer region via spacer sequences; recruitment of the dCas-activator complex: locates the target site of the dCas protein coupled with a transcription activator domain; supporting mechanisms for transcriptional stabilisation: Some engineered gRNAs can additionally recruit transcription factors. Therefore, engineering gRNAs can effectively enhance the efficiency of CRISPRa systems, enabling precise regulation of gene expression (Moon et al., 2019; Dong C. et al., 2022). Currently, one of the common methods used to maximize the functionality of CRISPR/Cas9 systems is the modification of gRNA spacer sequences. Spacer sequences are responsible for recognizing target DNA and have unique secondary structures, the ability of gRNA’s secondary structure to alter the activity of various CRISPR systems offers crucial direction for research on specificity optimization (Mullally et al., 2020).

The effectiveness of gene activation in mice is greatly increased by designing and optimizing circular gRNAs for Cas12f, with an improvement ranging from approximately 1.9 to 19.2-fold (Zhang et al., 2025). Activation potency is greatly increased by inserting RNA adapters (e.g., MS2, PP7) into the scaffold to recruit additional activators (e.g., MS2-VP64).

The effectiveness of CRISPRa systems is also influenced by target site selection and gRNA length. Kiani et al. discovered that the dCas9-VPR fusion protein could efficiently carry out transcriptional activation when sgRNA guide sequences of 10-20nt were used (Kiani et al., 2015). However, Cas9-mediated gene editing function was substantially eliminated when the guide sequence length dropped below 18 nt, indicating that transcriptional regulation exhibits greater tolerance to variations in guide sequence length than gene editing capacity.

Activation efficiency is influenced by the gRNA’s binding location; gRNAs that are close to the transcription start site (TSS) have more potent activation effects. Furthermore, gRNAs that target the sense strand are typically more successful than those that target the antisense strand (Piatek et al., 2015a). Stability and activation levels can be greatly increased by designing multiple sgRNAs that target the same promoter (Chavez et al., 2016; Gong et al., 2020).

### 2.3 Transcription activator

The most popular transcription activation domain is VP64, which is derived from the herpes simplex virus and consists of four VP16 units (amino acid sequence: DALDDFDLDML) joined by flexible linker peptides (GS). Compared to VP16, the multi-copy VP64 enhances stability in binding to transcription factors through multivalent interactions, significantly improving transcriptional activation efficiency (Chi et al., 1995), P65 is a mammalian transcription activation domain, and VP64 and P65 are commonly used as effectors to activate gene expression in eukaryotic cells, Rta is derived from the Epstein-Barr virus R transactivator, by tandemly fusing VP64 with p65 and Rta, a triple artificial transcription activation domain VPR was constructed, demonstrating significantly enhanced gene activation capacity compared to using VP64 alone (Guo et al., 2017). Therefore, in CRISPRa systems, tandem structures of multiple transcription activators can be employed to enhance efficient expression of target genes. However, for gene expression across different species, distinct combinations of dCas proteins and activation domains require separate optimization.

## 3 Advances in gene activation editing technology

**Figure 1:**
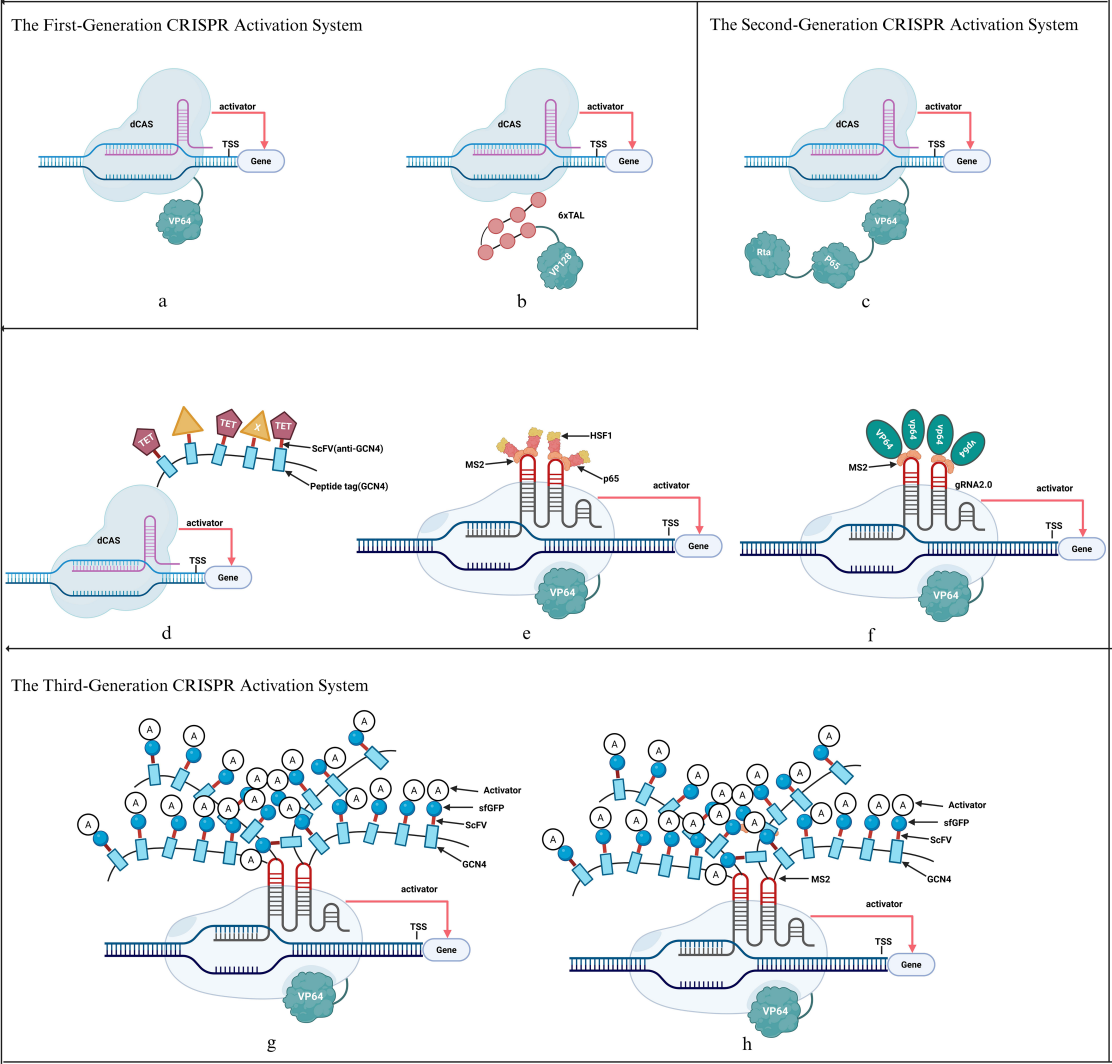
Schematic diagram of the development of different types of CRISPR transcription activation system. **(a)** dCas9-VP64, **(b)** dCas9-TV, **(c)** dCas9-VPR, **(d)** CRISPRa-SunTag, **(e)** CRISPRa-SAM, **(f)** CRISPR-Act2.0, **(g)** CRISPR-Act3.0, **(h)** CRISPR-Combo.

From basic transcription activation (VP64, p65) to enhanced transcription activation (SunTag system, dCas9-VPR system) and synergistic activation mediator systems (SAM system), CRISPR transcription activation strategies used in crops have undergone a number of advancements to date (**Figure 1**).

### 3.1 The first-generation CRISPR activation system

#### 3.1.1 dCas9-VP64

First-generation CRISPR activation methods in eukaryotic cells usually involve the direct fusion of catalytically inactive dCas9 proteins with transcription activation domains like p65 and VP64 (Maeder et al., 2013). This method has the ability to upregulate genes that are currently expressed as well as activate reporter or silenced endogenous genes (Kemaladewi et al., 2019). However, dCas9-VP64 shows very low activation effects in both bacteria and mammalian cells when targeting with a single guide RNA (gRNA), with an average activation fold of roughly 2–6 times (Kemaladewi et al., 2019; Ye et al., 2019; Javaid et al., 2021). This method also shows considerable activation potential in plant systems. For example, it can activate the uridine diphosphate glucose flavonoid glycosyltransferase (*UFGT*) gene in grapevines with an efficiency of 1.6-5.6 times (Ren et al., 2022). Activation effects can be improved by using numerous gRNAs for targeting. In *Arabidopsis thaliana*, for example, it effectively increased the expression of the *AtPAP1* and *miR319* genes by 2–7 times (Lowder et al., 2015), while in *Nicotiana benthamiana*, it increased the expression of the luciferase reporter gene that is regulated by the *NOS* promoter by 1-2.5 times (Vazquez-Vilar et al., 2016). To further enhance efficacy, researchers developed analogues of dCas9-VP64, such as dCas9-EDLL and dCas9-TAD (transcription activation domain). Transient expression experiments demonstrated that both analogues significantly activated the expression of the Bs3::*uidA* reporter gene (up to 14-fold), and appropriately increasing the number of gRNAs further elevated the activation levels (Piatek et al., 2015a).

#### 3.1.2 dCas9-TV

The dCas9-TV system is an enhanced and more effective variant. The dCas9-6TAL-VP128 complex is made up of two VP64 dimers that have been further coupled with six transcription activator-like effector (TAL) domains. In comparison to the base system, this system exhibits significantly improved activation capabilities across species: it activated the *WRKY30* gene in *Arabidopsis thaliana* by 139 times, whereas dCas9-VP64 was almost ineffective; it activated the *GW7* and *ER1* genes in rice by 79 and 62 times, respectively; and it activated the *ASCL1* and *OCT4* genes in human HEK293T cells by 46 and 14.6 times, respectively (Li et al., 2017).

Compared to dCas9-VP64, the dCas9-TV system also demonstrated higher efficiency in activating *UFGT* and the cold-response transcription factor gene *CBF4* in grapevines (Ren et al., 2022). Notably, when dCas9-TV simultaneously targeted *OsGW7* and *OsER1* genes in rice, a single sgRNA achieved up to 3,738-fold expression upregulation, with this activation effect stably inherited to the T3 generation (Xiong et al., 2021). Based on this, the optimized dCas9-TV system has been applied to crop improvement. For example, it effectively produced new germplasm with high yield, long fibers, and stress tolerance by focusing on the activation of three important genes in cotton: *GhKAO2* (gibberellin synthesis), *GhEXO2* (fiber elongation), and *GhSOS3* (salt stress response transcription factor) (Yu et al., 2023). This transcription activation method is equally successful in the field of disease resistance, increasing the expression of several defense-related genes in cowpea (*Pv-lectin* increased 6.97-fold, *Pv-thionin* increased 5.7-fold, and *PvD1* increased 1.37-fold) (Maximiano et al., 2025).

### 3.2 The second-generation CRISPR activation system

#### 3.2.1 dCas9-VPR

To accomplish more effective activation, researchers have tried to assemble several transcription activators onto a single dCas9-gRNA complex. VP64, p65AD, and Rta47 are three transcription activators whose activation domains were fused into a tandemly organized triple-effect molecule (VPR) and then integrated with dCas9 to create the dCas9-VPR system. The technique has been successfully used in a variety of cell types, including human, mouse, and Drosophila, and the results show that it effectively promotes gene expression (Chavez et al., 2015; Böhm et al., 2020; Liu et al., 2023). It can increase endogenous gene expression at the mRNA level by roughly 22–320 times in comparison to the dCas9-VP64 fusion protein, which employs a single transcription factor.

#### 3.2.2 CRISPRa-SunTag

Several tandemly repeated short peptides GCN4 (usually 4–20 copies) make up the second-generation amplification system SunTag. Each GCN4 unit can bind to a single-chain antibody scFv selectively, resulting in highly effective signal cascade amplification. Tanenbaum et al. initially created this technique to improve the CRISPRa systems’ transcriptional activation efficiency. SunTag’s ability to engage transcription activation domains (TADs) is greatly enhanced by attaching it to the N- or C-terminus of the dCas9 protein. The SunTag technology significantly improves gene activation efficiency as compared to conventional CRISPRa-VPR systems (Tanenbaum et al., 2014), compared to the direct fusion system dCas9-VP64, SunTag possesses the ability to simultaneously recruit multiple transcription factors and regulatory elements, thereby enabling the synergistic action of multiple transcription regulators to synchronously activate multiple genes.

Currently, the integration of the SunTag signal amplification system with CRISPRa technology has been widely applied across diverse biological systems including mammals, plants, and *Saccharomyces cerevisiae (*Ji et al., 2016; Papikian et al., 2019). Furthermore, the research team has also focused on integrating the CRISPR activation system with other signal amplification strategies. SunTag is not only applicable for gene activation, live-cell imaging, and DNA methylation editing, but can also be co-integrated with VP64 onto base editors to effectively enhance base editing efficiency (Dong X. et al., 2022).

Target RNA can be anchored onto at the RNA level by dCas13a linked with a single-chain antibody scFv that binds SunTag. Multiple scFv-GCN4 complexes can be recruited by a single dCas13a molecule, resulting in main signal amplification. Endogenous RNA has been effectively localized and imaged using the dCas13a-SunTag-BiFC system built on this concept (Chen et al., 2022).

The dCas9-SunTag-VP64 system effectively and selectively activates latent HIV-1 proviruses in humans by aggregating numerous VP64 transcription activation units using SunTag’s multivalent recruiting capacity (Ji et al., 2016). Additionally, researchers created a new inducible CRISPR activation tool called ER-Tag by combining the sgRNA expression module in SunTag with the β-estradiol-inducible XVE system. This tool has successfully achieved multi-gene coordinated regulation in plants including Arabidopsis, alfalfa, strawberry, and sheepgrass (Zhang et al., 2024a).

Based on the flexible SunTag architecture, dCas12a can also be fused with the 10×GCN4 peptide and utilize far-red light to induce expression of the scFv-p65-HSF1 activator, which is then recruited to the dCas12a-GCN4 complex. The resulting far-red light-inducible CRISPR-dCas12a system, FIdCA, achieved over 100-fold gene activation efficiency in mouse models (Wang et al., 2021).

Steven et al. used a SunTag VP64 transcriptional activation system built with dCas9-10×GCN4 and scFv-sfGFP-VP64 to successfully activate the Arabidopsis *FWA* gene in plant epigenetic regulation. This led to a late-flowering phenotype by reducing CG methylation levels in its promoter region (Papikian et al., 2019). Additionally, the human genome’s long terminal repeat sequence *LTR12C* was activated using the Cas9-SunTag-VP64 and dCas9-SunTag-p300 systems, which controlled the expression of adjacent protein-coding genes (Ohtani et al., 2024).

The improved scFv shows strong affinity and specificity for the GCN4 epitope in molecular design. Large numbers of effector proteins can be recruited to the target genomic locus when the scFv-effector protein fusion is produced intracellularly because numerous scFs can bind to multiple GCN4 sites on a single SunTag scaffold at the same time. When it comes to activating endogenous genes like *ASCL1* and *OCT4* as well as human reporter genes, this method is far more effective than the conventional dCas9-VP64 direct fusion system (Tanenbaum et al., 2014).

##### 3.2.2.1 MoonTag

Casas-Mollano, J A. et al. created the MoonTag nanobody-peptide interaction system in order to solve the instability of scFv expression in plants inside the SunTag system. In this system, *NbGP41* nanobodies are fused to VP64 and dCas9 is coupled with multiple repetitions of the GP41 peptide generated from HIV gp41. MoonTag demonstrated superior activation efficiency in plants compared to SunTag, exhibiting stable expression and broad-spectrum activity (Casas-Mollano et al., 2023).

##### 3.2.2.2 CRISPRa-SAM

(Synergistic Activation Mediator): CRISPRa-SAM system is a highly efficient transcriptional activation tool based on CRISPR-dCas9 (Konermann et al., 2015). Three parts make up the entire system: (1) a fusion protein of dCas9 and the VP64 transcription activation domain; (2) a modified sgRNA (also called Scaffold RNA) with two RNA hairpin structures on its scaffold that bind to the MS2 phage coat protein (usually found in the tethered loop region and/or stem-loop 2 region of tracrRNA) (Chavez et al., 2016); and (3) the activation co-activator fusion protein MCP-p65-HSF1 (MPH), which specifically recognizes the MS2 hairpin on the sgRNA and HSF1 to the target site (Mali et al., 2013). When these three elements are co-expressed, the MCP-p65-HSF1 fusion protein is recruited by the MS2 hairpin and dCas9-VP64 is guided by the designed sgRNA to attach to particular genomic locations via complimentary sequences. This assembly forms a complex containing multiple activation domains at the target promoter region, enabling synergistic and highly efficient transcriptional activation. This system has demonstrated broad applications in gene function research and disease therapy. For example, in mature adipocytes, CRISPRa-SAM-mediated intervention markedly increased important adipogenesis genes like *Pparγ2* (up to 104-fold) and *Ucp1* (up to 4×10³-fold) (Lundh et al., 2017). Furthermore, the dCas9-SAM system has been developed as a novel tool for reversing HIV latency, offering the potential for permanent clearance of the HIV-1 latent reservoir (Zhang et al., 2015), and holds significant promise in fields such as cancer therapy (Xu et al., 2025). More research is necessary to fully understand the potential applications of the dCas9-SAM system in crops.

##### 3.2.2.3 CRISPR-Act2.0

Lowder et al. developed a system based on dCas9-VP64, incorporating the gRNA 2.0 scaffold and recruiting the MS2-VP64 fusion protein. Through the T2A peptide chain, this system enables co-expression of dCas9-VP64 and MS2-VP64, supporting multi-gRNA assembly to simultaneously target multiple gene promoters. In *Arabidopsis thaliana*, CRISPR-Act2.0 dramatically increases activation efficiency, increasing *FIS2* expression by up to 1,500 times and *PAP1* expression by 30–45 times. It also shows applicability in monocotyledons (Lowder et al., 2018).

### 3.3 The third-generation CRISPR activation system

#### 3.3.1 CRISPR-Act3.0

The key elements are dCas9-VP64, which is a fusion of the VP64 activation domain; gR2.0 sgRNA scaffold, which has two MS2 RNA adapters to recruit MCP proteins; 10xGCN4SunTag, which is fused to MCP to improve activator recruitment capacity; and 2xTAD activator, a recently created transcription activation domain that greatly increases activation efficiency. CRISPR-Act3.0 achieves 4-6-fold higher activation efficiency than second-generation systems in rice protoplasts for multiple genes, with *OsGW7* and *OsER1* activation exceeding 250-fold and 100-fold, respectively, supports simultaneous activation of up to seven genes in synthetic pathways. Notably, the dCas12b system can recognize VTTV PAM sequences when the CRISPR-Act3.0 method is applied, which makes it appropriate for AT-rich promoters. The developed dSpRY-Act3.0 system may target a variety of PAM types and shows almost negligible PAM constraints (Pan et al., 2021). Using sgRNAs made for several endogenous genes undergoing transient expression and stable transformation studies, the CRISPR/Cas3.0 system was used in *Dendrobium officinale*. Transient activation of *MCT* significantly increased dendrobine content. Dendrobium alkaloid content increased by up to 35.6% in transgenic plants after stable transformation using sgRNAs targeting genes like *MCT*, *STR1*, *CYP94C1*, and *HMGR*, demonstrating that CRISPR/Act3.0 exhibits more pronounced effects in low-content species (Zhao et al., 2025).

#### 3.3.2 CRISPR-Combo

The MS2 hairpin structure of the CRISPR-Combo system, which was first created in rice, attracts the transcription activation complex MCP-SunTag-2xTAD. It allows for simultaneous gene activation and genome editing, as evidenced by the notable activation of *SISFT* in tomatoes (Byiringiro et al., 2024).

Summarizing the abovementioned activation systems reveals that SunTag, dCas9-VPR, and dCas9-TV primarily achieve transcriptional activation by modifying the dCas9 protein. In contrast, SAM, CRISPR-Act2.0, and CRISPR-Act3.0 systems mainly enhance activation capacity through engineered gRNA design. urther analysis and new experimental data are needed to determine whether these different approaches may effectively increase target gene expression in plant systems.

## 4 Application of gene activation editing technology in crops

**Figure 2:**
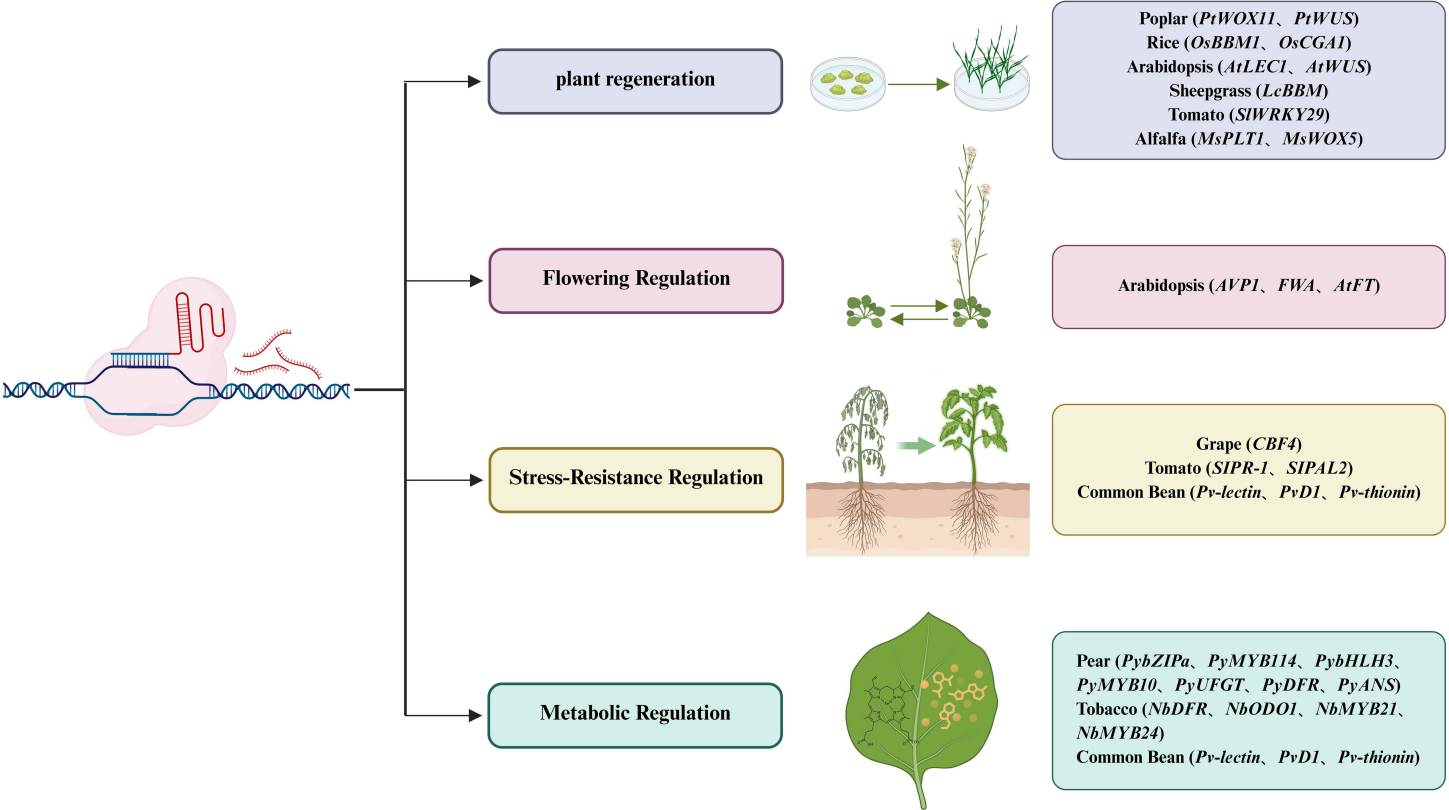
Application of CRISPR transcription activation system in crop trait improvement.

With the development of various CRISPR-dCas gene activation systems, their application as tools across diverse research fields has grown increasingly widespread (**Figure 2**). The main applications of these activation techniques are in metabolism, stress-resistant breeding, flowering regulation, and plant regeneration (**Table 1**). Currently rice is the most extensively studied crop using CRISPRa technology, with its application primarily focused on regulating key agronomic traits such as yield and stress tolerance. For instance, activating the endogenous gene *OsGW7* via CRISPRa results in a more elongated grain shape, thereby enhancing both visual quality and yield. In terms of stress tolerance, rice drought resistance is increased by activating the *OsER1* gene, which controls stomatal development (Xiong et al., 2021). Notably, compared to traditional methods, CRISPRa demonstrates unique advantages in enhancing stress resistance. For instance, activating the Arabidopsis *AtAREB1* gene using the CRISPRa-VPR system resulted in significantly improved drought tolerance without the negative effects of traditional overexpression, such as growth retardation. This offers a novel strategy for balancing stress resistance with growth and development (Roca Paixão et al., 2019). Moreover, CRISPRa has demonstrated significant potential in addressing challenges related to crop genetic transformation. Many superior crop varieties face difficulties in tissue culture regeneration, severely limiting the application of gene editing technologies. To overcome this bottleneck, researchers employed CRISPRa technology to precisely activate endogenous regeneration-related genes (such as *WUS*), achieving a significant increase in callus regeneration efficiency even in traditionally non-regenerative species like tomatoes. This innovative strategy has been successfully applied to various plants with lower regenerative capacity, including Arabidopsis, poplar, pepper, soybean and sugarcane (Shabbir et al., 2026), significantly expanding the scope of gene editing technology in crop improvement.

**Table 1:** The application of various CRISPR-dCas gene activation systems in plants.

Species	Gene	Editing system types	Year	Phenotype	Fold increase	References
*Arabidopsis thaliana*	AtPAPI	dCas9-VP64	2015		2-7	(Lowder et al., 2015)
	miR319				3-7.5	
	AIFIS2				400	
*Nicotiana benthamiana*	NbPDS	dCas9-EDLL	2015		3.5	(Piatek et al., 2015b)
	AtPDS	dCas9-TAL			4	
*Nicotiana benthamiana*	pNOS::LUC	dCas9-EDLL	2016		2.2	(Vazquez-Vilar et al., 2016)
	pNOS::LUC	dCas9-VP64			2.3	
*Arabidopsis thaliana*	PAP1	dCas9-VP64 + p65-HSF	2017	Leaves turn purple in bright light, double-targeted lines show abnormal phenotypes such dwarfism	2-3	(Park et al., 2017)
	AVP1			Increased number of leaves, larger leaf area, delayed flowering, and enhanced drought tolerance	2-5	
*Arabidopsis thaliana*	LexA	dCas9-VP64	2017		2.4	(Li et al., 2017)
*Arabidopsis thaliana*	PAP1	CRISPR-Act2.0	2018		30–45	(Lowder et al., 2018)
	FIS2				1500	
	ULC1				40	
	miR319				6	
Oryza sativa L.	Os03g01240			protoplast	3	
	Os04g39780				4	
	Os11g35410					
*Arabidopsis thaliana*	AREB1	CRISPRa/dCas9-HAT	2019	Dwarfing	1.7-2	(Roca Paixão et al., 2019)
	RD29A				2.6-3	
*Nicotiana benthamiana*	NbDFR	CRISPR–dCasEV2.1	2019		~10,000	(Selma et al., 2019b)
	NbAN2				>4,000	
	pSlDFR::Luc				90-400	
	pNos::Luc				3-13	
*Arabidopsis thaliana*	FWA	SunTag VP64	2019	delayed flowering		(Papikian et al., 2019)
	ATR					
	AP3				>500	
	CLV3			stem cell abnormality		
Oryza sativa L.	OsER1	dAaCas12b-TV + Aac.3 + MS2-VPR	2020		5–8	(Ming et al., 2020)
	OsGW7					
Zea mays L.	TrxH	dCas9-VP64	2020		2	(Gentzel et al., 2020)
	PDS1				2.5	
*Arabidopsis thaliana*	FWA	SunTag-VP64	2020	delayed flowering	2.3-78	(Ghoshal et al., 2020b)
Oryza sativa L.	OsCGA1	dCas9-VP64-EDLL	2021	Enhancing Chloroplast Development in Oryza sativa L. BS Cells	2-5	(Lee et al., 2021)
*Arabidopsis thaliana*	AtFT	CRISPR-Combo	2022	Extra early flowering		(Pan et al., 2022)
hybrid poplar	PtWOX11			Enhance regenerative capacity	up to 800 times	
	PtWUS			Accelerated Regeneration	100-200	
Oryza sativa L.	OsBBM1			Significantly enhance regeneration rates and achieve hormone-free regeneration		
*Pyrus communis*	PybZIPa	CRISPR-Act3.0	2022	enhanced anthocyanin accumulation	40	(Ming et al., 2022)
	PyMYB114 + PybHLH3			enhanced anthocyanin accumulation	10-20	
	PyMYB10 + PybHLH3				2-6	
	PyUFGT			enhanced anthocyanin accumulation	10-40	
	PyDFR			enhanced anthocyanin accumulation	2-10	
	PyANS			enhanced anthocyanin accumulation	2-10	
Oryza sativa L.	OsGW7	CRISPR-Act3.0	2022	protoplast	>250	(Pan and Qi, 2022)
	OsER1				>100-250	
	OsBAM1				1.3–24	
	OsTPR-like				~60	
	OsCCR1				~20	
	OsCHS				>30	
	OsCHI				<10	
	OsF3H				>30	
	OsF3’H				>30	
	OsDFR				>30	
	OsLAR				2–8	
	OsRc				40	
	OsTTG1				40	
	OsDXS, OsPDS, OsPSY, OsCRTISO, OsZISO, OsZDS, OsLCYB				10–20	
*Arabidopsis thaliana*	AtFT			early flowering	80-500	
	AtTCL1			Reduction in leaf trichomes	3–20	
*Solanum lycopersicum*	SFT			protoplast	20-240	
*Nicotiana benthamiana*	NbDFR	dCasEV2.1+pPVX_VIGR	2022		11,000	(Selma et al., 2022b)
	NbODO1			Accumulation of non-volatile metabolites	>600	
	NbMYB21				>110	
	NbMYB24				>1200	
Vitis vinifera L.	UFGT	dCas9-VP64	2022		5.6	(Ren et al., 2022)
	UFGT	dCas9-TV			5.7–7.2	
	CBF4	dCas9-TV		Enhanced cold tolerance	19.3–42.3	
Oryza sativa L.	OsER1	Cas12j2 + 67AL–VP128	2022		4-10	(Liu et al., 2022)
	OsNRT1.1A				2-4	
*Nicotiana benthamiana*	NbDFR	CI/dCasEV2.1	2022		2600	(Garcia-Perez et al., 2022)
	NbPAL2				245	
Stevia rebaudiana Bertoni	UGT76G1	CRISPR/dCas9-VP64	2022		18.39-27.51	(Ghose et al., 2022)
*Solanum lycopersicum*	SIPR-1	dCas9-SET and dCas9-VP64	2023	Enhanced disease resistance without compromising key agronomic traits	40-110	(García-Murillo et al., 2023)
Oryza sativa L.	OsmIR528/OsNRT1.1A/OsWx	dLrCas9-TV	2023	protoplast	2-3	(Zhong et al., 2023)
	OsmIR528	LrCas9-TV			19	
	OsWx				150	
*Setaria viridis*	SvCLV3	MoonTag	2023		50-100	(Casas-Mollano et al., 2023)
*Arabidopsis thaliana*	AtFT				20-50	
	AtCLV3				100-350	
*Solanum lycopersicum*	SIPAL2	CRISPR/dCas12a-SET(LbCpf1)		lignin accumulation has been strengthened and disease resistance has been greatly increased	6.5	(Rivera-Toro et al., 2025)
hybrid poplar	TPX2	CRISPR-Act3.0	2023		1.2-2.9	(Yao et al., 2023)
	LecRLK-G				1.9-7	
*Arabidopsis thaliana*	LEC1	ER-Tag	2024	Significantly accelerates root regeneration	>10,000	(Zhang et al., 2024b)
	WUS				up to 4000	
*Medicago sativa*	MsPLT1			Promote callus and shoot formation		
	MsWOX5					
Fragaria vesca L.	FveGRF-FveGIF					
*Leymus chinensis*	LcBBM			Improve regeneration efficiency		
Phaseolus vulgaris	Pv-lectin	dCas9-TV	2025	Enhance antimicrobial peptide synthesis capacity	6.97	(Maximiano et al., 2025)
	PvD1				1.37	
	Pv-thionin				5.7	
*Arabidopsis thaliana*	AtFT	CRISPR-Act3.0	2025	early flowering	>50	(Cheng et al., 2025)
*Solanum lycopersicum*	SlWRKY29	dCas9-SET-MS2-SET	2024	High seedling yield, early rooting	3-4.4	(Valencia-Lozano et al., 2024)
		dCas12-SET		High efficiency in shoot and root induction, robust plant growth	3.8-6	

The *FWA* and *AtFT* genes were tested using the SunTag, CRISPR-Combo, and CRISPR-Act3.0 systems in order to regulate flowering time. The results showed high activation efficiency with different late-flowering and early-flowering phenotypes, respectively (Papikian et al., 2019; Ghoshal et al., 2020a; Pan et al., 2021; Pan et al., 2022; Cheng et al., 2025). This provides robust support for shortening breeding cycles.

CRISPR-Combo (poplar, rice) and ER-Tag (alfalfa, sheepgrass) both exhibited gene activation efficiencies ranging from 100 to 4000-fold in plant regeneration and genetic transformation, greatly increasing regeneration rates (Pan et al., 2022; Zhang et al., 2024a). The dCas9-SET-MS2-SET and dCas12-SET systems demonstrated strong somatic embryo induction and rooting efficiency for tomatoes, increasing efficiency by three to six times (Valencia-Lozano et al., 2024). These methods offer prospective means of improving transformation success rates and regeneration efficiency in resistant species.

The dCas9-SET, dCas9-VP64, and CRISPR/dCas12a-SET (LbCpf1) systems all markedly enhanced the expression levels of tomato *SIPR-1* (40-110-fold) and *SIPAL2* (6.5-fold) in terms of stress response regulation, improving disease resistance without altering agronomic features (García-Murillo et al., 2023; McLaughlin et al., 2025; Rivera-Toro et al., 2025). The dCas9-TV system enhances antimicrobial peptide synthesis in common beans by increasing *Pv-lectin*, *PvD1*, and *Pv-thionin* expression (Maximiano et al., 2025), and also boosts cold tolerance in grapevines by elevating *CBF4* expression (Ren et al., 2022).These studies reveal that merely doubling or tripling the expression levels of certain genes can induce significant phenotypic changes. This suggests biological systems may exhibit high sensitivity to the expression levels of specific genes.

CRISPR activation technology was used to precisely increase plant metabolic pathways. In *Dendrobium officinale*, the CRISPR-Act3.0 system simultaneously activated multiple endogenous genes involved in dendrobine biosynthesis (such as *MCT*, *CMAO*, and *BGLU*), successfully increasing dendrobine yield by 30.1% (transient transformation) and 35.6% (stable transgenic lines) (Zhao et al., 2025). In Arabidopsis, the endodermis-specific CRISPRa-SunTag system precisely reconfigured metabolic pathways by simultaneously activating six flavonoid synthase genes, successfully restoring flavonoid accumulation in mutant plants to wild-type levels (Houbaert et al., 2025). The dCasEV2.1 system, delivered via pPVX_VIGR infection in tobacco, substantially increased non-volatile metabolite accumulation (Selma et al., 2019a). These investigations show that CRISPRa technology offers effective instruments for studying complex plant natural products in synthetic biology.

## 5 Various methods for introducing CRISPRa into plants

CRISPRa components must be successfully delivered into plant cell nuclei to function effectively. Delivery represents the primary bottleneck in CRISPRa applications. Currently, CRISPRa introduction into plants primarily involves the following five approaches:

### 5.1 Agrobacterium-mediated transformation

Agrobacterium-mediated genetic transformation represents the most mature technology in plant genetic engineering. It allows for the delivery of big DNA fragments and provides a steady transformation efficiency. Combining techniques such as the addition of the regeneration-promoting factor *CaREF1*, the RUBY visual reporter system, and optimized genotype screening can significantly increase transformation efficiency for crops that are challenging to transform, such as chili peppers (Li et al., 2003).

### 5.2 The gene gun bombardment

The gene gun is the recommended transformation technique for monocotyledons (such as wheat and maize) and some woody plants that are challenging to transform using Agrobacterium, it can deliver large DNA fragments (Vollen et al., 2025), Plasmid DNA encoding the entire complement of components can be delivered to CRISPRa. Although the gene gun method and agrobacterium-mediated transformation based on DNA-stable genetic transformation are still widely used, they are subject to regulatory restrictions when it comes to integrating exogenous DNA.

### 5.3 Delivery of Ribonucleoproteins

Ribonucleoprotein delivery refers to the pre-assembly of *in vitro* purified Cas fusion proteins with *in vitro* transcribed gRNAs into complexes, which are then directly introduced into plant cells. The editing elements are rapidly degraded by endogenous proteases and RNases after completing their function, preventing integration into the genome. The final product contains no exogenous DNA, but its delivery efficiency relies on PEG-mediated protoplast transfection, gene gun bombardment, emerging nanocarrier technologies, and cell-penetrating peptide techniques (Chang et al., 2022).

### 5.4 Viral vector-mediated delivery

Viral delivery eliminates the need for tissue culture, enables systemic delivery, and features a short operational cycle. For CRISPRa, virus-mediated transient activation is particularly well-suited for metabolic engineering (Selma et al., 2022a). However, RNA viral vectors have limited capacity, making it difficult to accommodate larger dCas9-activator fusion protein sequences; viral infection may interfere with endogenous gene expression in plants; and biosafety regulatory requirements may restrict the field application of viral vectors (Shen et al., 2024).

In summary, appropriate technical choices must be made in research by considering the biological characteristics of the target species, the experimental objectives, and regulatory requirements.

## 6 Challenges and prospects

Despite providing strong tools for plant breeding, CRISPRa technology still has many biological and technical barriers to widespread use. These limitations primarily center on activation efficiency, target selection, and delivery systems:

1. Uneven activation efficiency: in current CRISPRa applications, the main barrier is uneven activation efficiency. Different genes exhibit significant variability in their response to the CRISPRa system, even when very effective activation structures like VPR or SunTag are used, certain genes may only see a 2–3 fold increase in expression or not activate at all, while others may experience up to a thousandfold upregulation (Lowder et al., 2018). Overall, CRISPRa demonstrates superior activation efficacy for genes with low to moderate baseline expression levels, however, it frequently fails to overcome chromatin-level repression in highly methylated or profoundly silenced genes. Furthermore, the complex and diverse structures of plant promoters further complicate precise activation, the ideal region for sgRNA targeting is usually located between -50 and -500 bp upstream of the transcription start site, however, if this region is located within densely packed chromatin areas or lacks appropriate PAM sequences (such as NGG), activation effectiveness drops significantly.
2. Off-target transcription and non-specific activation risks: Unlike the off-target effects of traditional CRISPR cleavage systems, enhancer and promoter elements in plant genomes often exhibit regional regulatory properties, simultaneously influencing the expression of multiple adjacent genes. After binding to the target site, the CRISPRa complex may recruit transcription machinery (such as RNA polymerase) that inadvertently activates other genes surrounding the target, leading to unintended phenotypic changes. Even when using high-fidelity dCas9 variants, binding to other genomic locations remains possible. Although DNA cleavage does not occur, recruitment of transcription activators at wrong sites may mildly disrupt the genome-wide transcriptional network, triggering global expression perturbations (Wada et al., 2022).
3. Vector size constraints and delivery efficiency issues: CRISPRa systems are significantly larger in physical size than traditional CRISPR systems. The traditional Cas9 gene is approximately 4 kb, while the dCas9 protein itself has a molecular weight of 160 kDa, further increasing in size when fused with a transcription activation domain. This increase in size not only complicates vector construction but also reduces its delivery efficiency into plant cells. Furthermore, many viral vectors suitable for plants (commonly used for transient expression) impose strict loading capacity constraints. The oversized CRISPRa components struggle to package into a single viral particle, thereby limiting the application of efficient viral-based delivery systems. Simultaneously, increased plasmid size typically reduces genomic integration efficiency when employing methods such as Agrobacterium-mediated transformation or the gene gun technique (Lowder et al., 2017).
4. Coordinating polygenic activation is challenging. Gene silence may result from recurrent promoter usage in polygenic stacking (Zhao et al., 2025), coordinated polygenic activation requires well-defined pathways and synthetic routes. Additionally, the activation effectiveness of individual genes may decline as the number of activated genes rises, forcibly and continuously activating a particular endogenous gene may disrupt metabolic balance within the plant. For instance, while persistent activation of immune genes enhances disease resistance, it typically results in stunted growth and reduced yield.
5. Chromatin environment and epigenetic barriers: the local chromatin state surrounding the target gene severely limits the CRISPRa system’s activation efficiency. For genes located in highly condensed heterochromatic regions (e.g., near centromeres) or those under stringent epigenetic regulation such as heavy DNA methylation, their promoter regions are typically in a physically occluded or highly repressed state. This significantly reduces the efficacy of transcriptional activation by impeding the dCas9 activation complex’s efficient binding and residency.
6. Persistence issues: traditional CRISPR knockout technology allows for the removal of the Cas9 element through genetic segregation in subsequent generations, leaving only the mutation at the target site and yielding “non-transgenic” edited plants. In contrast, CRISPRa systems typically require the persistent presence of the dCas9-activator complex within cells to maintain gene upregulation; removal of this transgene often causes target gene expression returns to normal levels. Recent innovations in epigenetic editing aim to achieve durable and heritable gene activation, but their long-term stability and genetic reliability within plant systems remain to be further validated.

Currently, in addition to CRISPRa, research has reported multiple synthetic genetic circuit activation systems. Synthetic genetic circuits enable precise regulation of multi-signal integration and complex expression patterns through Boolean logic operations (e.g., AND, OR, NOT), acting solely via transcriptional control without altering DNA sequences, thereby avoiding off-target cutting risks (Brophy et al., 2022). Furthermore, this system allows quantitative control of gene expression levels by adjusting the number and position of transcription factor binding sites within promoters or introducing site-directed mutations. However, synthetic genetic circuit systems also have the following limitations: their design and construction are relatively complex, typically requiring multiple rounds of optimization; the bacterial-derived DNA-binding domains they rely on may induce immune responses in plants; and for spatial regulation, they still require known tissue-specific promoters to drive transcription factors, failing to achieve precise localization entirely independent of endogenous elements. In contrast, the CRISPRa system offers greater regulatory flexibility by enabling spatiotemporal gene activation through diverse approaches, including tissue-specific promoters, chemical induction, or optogenetic tools. Overall, synthetic genetic circuit systems offer significant advantages in programmability and quantitative expression regulation, making them particularly well-suited for constructing complex artificial gene expression networks. In contrast, CRISPRa excels in versatility, ease of operation, and compatibility with endogenous regulatory systems. These two approaches can complement each other functionally in plant, and their future integration may further advance gene expression programming.

From a safety perspective, CRISPRa technology presents a dual-edged sword. On one hand, since the final product contains no foreign DNA fragments, its biological risks differ fundamentally from those of traditional genetically modified organisms (GMOs). However, changes in gene expression levels itself may result in phenotypic changes, and CRISPRa may still have unanticipated off-target effects. This necessitates safety assessments, with the heritability of CRISPRa’s epigenetic modifications also being a key regulatory focus. As a result, controlling CRISPRa crops poses particular difficulties. How to encourage the use of CRISPRa technology in crop production while maintaining safety will be the main goal of future regulations.

In the future, gRNA design techniques can be optimized to produce targeted, highly active gRNAs that target distinct genes by combining high-throughput phenotyping, omics data, and AI predictions. Plant transformation procedures will be made simpler by finding Cas proteins with improved selectivity and smaller sizes as well as creating effective delivery systems such viral vectors and nanomaterials.

## 7 Conclusion

**Figure 3:**
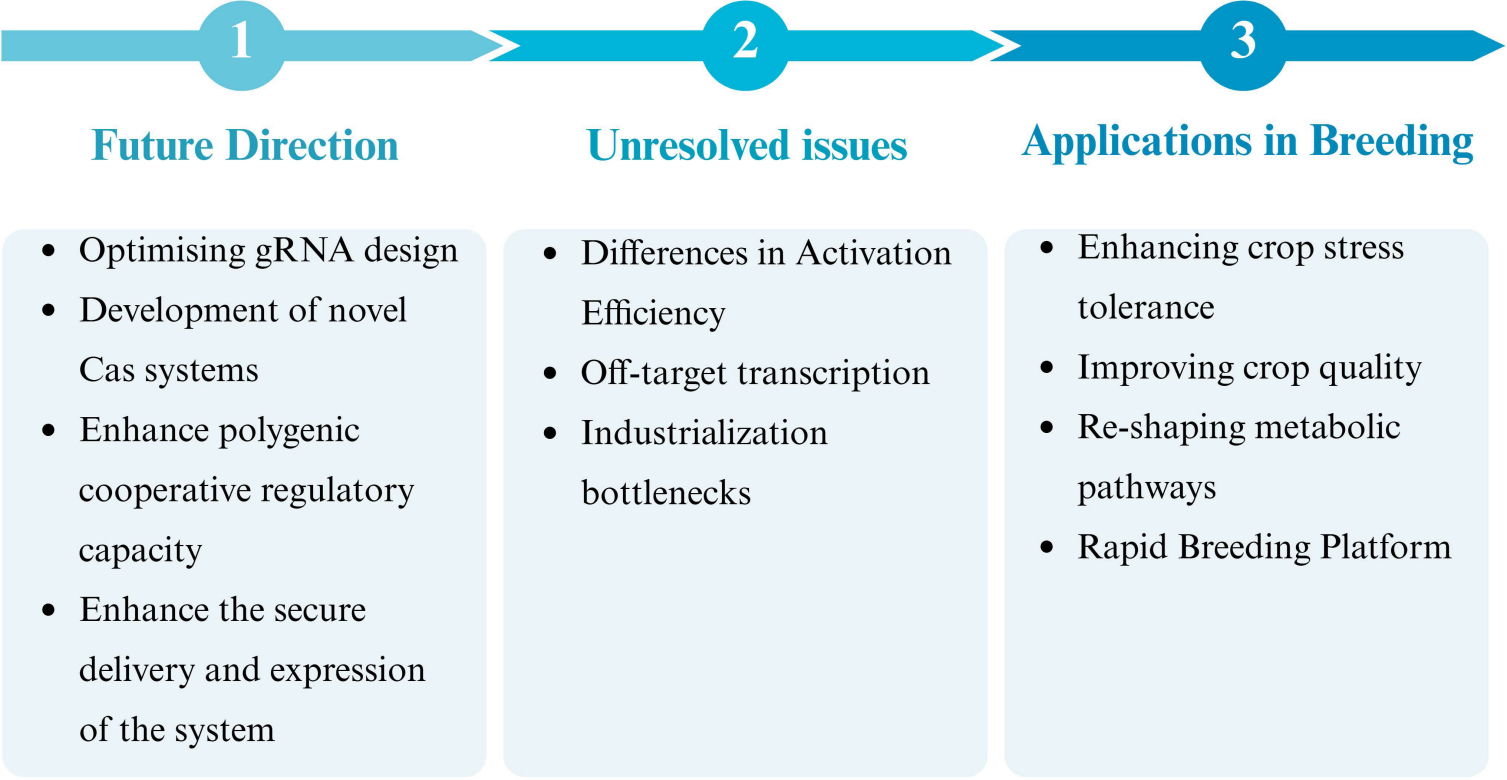
Future roadmap for CRISPRa technology applications in breeding.

CRISPRa, or CRISPR-based transcription activation systems, have developed into essential instruments for precisely regulating crop gene expression. These systems began with basic dCas9-VP64 fusion systems and have now developed into multi-component complexes such as dCas9-TV, dCas9-VPR, SunTag, SAM, CRISPR-Act 2.0/3.0, and CRISPR-Combo. These systems have achieved significant progress in enhancing gene activation efficiency, improving targeting specificity, and enabling multi-gene synergistic regulation. CRISPRa technology holds broad application prospects in regulating crop growth and development, enhancing stress resistance, improving quality traits, and rearranging metabolic pathways. Furthermore, integrating this technology with strategies such as inducible expression systems and viral vector delivery has expanded its practicality and flexibility in plant synthetic biology and crop molecular breeding. Currently, CRISPRa still faces the following challenges: activation efficiency varies across genes and species, potential off-target transcription risks exist, large vector delivery is difficult, and multi-gene co-regulation is complex (**Figure 3**). Future advancements may overcome these bottlenecks through AI- and multi-omics-assisted gRNA design, development of compact and efficient Cas protein variants, and establishment of secure delivery systems. It is expected to speed up the breeding of crop varieties that are stress-tolerant, high-yielding, and high-quality as this technology develops further and integrates deeply with emerging biotechnologies. This will significantly contribute to maintaining global food security and advancing sustainable agricultural development.
